# Supplementary material for: How Does Pseudo-Jahn-Teller Effect Induce the Photoprotective Potential of Curcumin?
Source: Molecules. 2023 Mar 25;28(7):2946. doi: 10.3390/molecules28072946 (PMC10096455; doi:10.3390/molecules28072946)
Supplement: Supplementary file 1 [file molecules-28-02946-s001.zip › molecules-2269826-supplementary.pdf]

How Does Pseudo-Jahn-Teller Effect Induce the Photoprotective Potential of Curcumine?

*Dagmar Štellerová, Vladimír Lukeš, Martin Breza **

Institute of Physical Chemistry and Chemical Physics, Slovak University of Technology in Bratislava, Radlinského 9, SK-812 37 Bratislava, Slovakia

Table S1. Kernel groups K(Γ, Λ) of the parent groups Γ for active coordinates of Λ symmetry (the preserved σ_v and σ_v‘ mirror planes are coincident with and perpendicular to the curcumin molecular plane, respectively) [S1]

**Γ**	**Λ**	**K(Γ, Λ)**
C_{2v}	a_1	C_{2v}
	a_2	C_2
	b_1	$C_s(\sigma_v)$
	b_2	$C_s(\sigma_v')$
C_s	*a’*	C_s
	a”	C_1

Table S2. Relations between the irreducible representations Λ_0 of the parent group Γ_0 and the irreducible representations Λ_1 of its 1st order subgroups Γ_1 (the preserved σ_v and σ_v' mirror planes are coincident with and perpendicular to the curcumin molecular plane, respectively) [S1]

Γ_0	Λ_0	Γ_1	Λ_1
C_{2v}	A_1	C_2	A
	A_2		A
	B_1		B
	B_2		B
C_{2v}	A_1	$C_s(\sigma_v)$	A'
	A_2		A''
	B_1		A'
	B_2		A''
C_s	A'	C_1	A
	A''		A

Table S3a. TD-M062X/cc-pVDZ electron transitions of stable keto-keto forms (model **Ia** and **Ib**) of C_2 symmetry. Excitation energies (E_{exc}) and corresponding wavelengths (λ) are in eV and nm, respectively. Oscillator strengths (*f*) are in atomic units.

	**Ia**			**Ib**		
**No.**	E_{exc}	λ	f	E_{exc}	λ	f
1	3.6161	342.87	0.0020	3.4843	355.84	0.0208
2	3.6880	336.18	0.0050	3.6986	335.22	0.0031
3	4.1536	298.50	1.3837	3.9600	313.09	0.9127
4	4.3402	285.67	0.0987	4.1899	295.91	0.3202
5	4.7143	263.00	0.0040	4.8129	257.61	0.0137
6	4.8637	254.92	0.0288	4.8201	257.22	0.0057
7	4.8867	253.72	0.0300	4.9446	250.75	0.0441
8	5.437	228.04	0.0003	4.9475	250.60	0.0711
9	5.6272	220.33	0.0116	5.5959	221.56	0.4586
10	5.6942	217.74	0.1302	5.6339	220.07	0.1887
11	5.7299	216.38	0.4482	5.6793	218.31	0.0295
12	5.9327	208.99	0.0041	6.0443	205.13	0.0287
13	5.9871	207.09	0.0044	6.1365	202.04	0.0278
14	6.2076	199.73	0.0753	6.1459	201.74	0.0071
15	6.3026	196.72	0.0667	6.3064	196.60	0.1111
16	6.4839	191.22	0.0860	6.3095	196.50	0.0653
17	6.5109	190.43	0.0100	6.4369	192.61	0.0020
18	6.7647	183.28	0.0002	6.4451	192.37	0.0003
19	6.7770	182.95	0.0010	6.7271	184.30	0.0006
20	6.7845	182.75	0.0007	6.7517	183.63	0.0001
21	6.8529	180.92	0.0059	6.8551	180.86	0.0023
22	6.8574	180.80	0.0053	6.8558	180.85	0.0006
23	6.8623	180.68	0.0023	6.9057	179.54	0.0125
24	6.8992	179.71	0.0006	6.9267	179.00	0.2578
25	6.9699	177.88	0.3410	6.9398	178.66	0.1069
26	6.9997	177.13	0.1948	6.9640	178.04	0.0002
27	7.0404	176.10	0.0106	6.9655	178.00	0.0074
28	7.1000	174.63	0.0037	6.981	177.60	0.0099
29	7.2425	171.19	0.0027	7.1148	174.26	0.0552
30	7.3600	168.46	0.0001	7.1341	173.79	0.0176

Table S3b. TD-B3LYP(SMD=water)/aug-cc-pVDZ//M062X/cc-pVDZ electron transitions of stable keto-keto forms (model **Ia** and **Ib**) of C_2 symmetry. Excitation energies (E_{exc}) and wavelengths (λ) are in eV and nm, respectively. Oscillator strengths (*f*) are in atomic units.

	**Ia**			**Ib**		
**No.**	E_{exc}	λ	f	E_{exc}	λ	f
1	3.3284	372.50	0.6482	2.9706	417.38	0.5374
2	3.3965	365.04	0.0643	3.1063	399.13	0.1117
3	3.4872	355.54	0.5009	3.5081	353.42	0.0172
4	3.5412	350.12	0.0951	3.6116	343.29	0.1143
5	3.7761	328.34	0.0014	3.6143	343.04	0.2495
6	3.8687	320.48	0.0005	3.8332	323.45	0.2490
7	4.0302	307.64	0.1799	3.8341	323.37	0.0260
8	4.1187	301.03	0.1691	3.9016	317.78	0.1592
9	4.3065	287.90	0.0026	4.4180	280.63	0.0207
10	4.3917	282.32	0.0034	4.4181	280.63	0.0381
11	4.4841	276.49	0.0019	4.4293	279.92	0.0060
12	4.5987	269.60	0.0013	4.8362	256.37	0.2299
13	4.8858	253.77	0.0699	4.9119	252.42	0.1159
14	4.9325	251.36	0.3240	4.9161	252.20	0.2014
15	5.0650	244.79	0.0014	5.0288	246.55	0.0281
16	5.0794	244.09	0.0011	5.0317	246.41	0.0552
17	5.2359	236.79	0.0032	5.0738	244.36	0.0013
18	5.2705	235.24	0.0006	5.0827	243.93	0.0004
19	5.3057	233.68	0.0063	5.2234	237.36	0.0066
20	5.3168	233.19	0.0807	5.2262	237.23	0.0013
21	5.3402	232.17	0.0065	5.2449	236.39	0.0188
22	5.3635	231.16	0.0191	5.2896	234.39	0.0001
23	5.4240	228.59	0.0673	5.3569	231.45	0.0011
24	5.4325	228.23	0.0208	5.3839	230.29	0.0003
25	5.4806	226.22	0.0381	5.5182	224.68	0.0010
26	5.5041	225.26	0.0536	5.5376	223.89	0.0020
27	5.5167	224.74	0.0091	5.5471	223.51	0.0406
28	5.5245	224.42	0.0113	5.5665	222.73	0.0227
29	5.5782	222.27	0.0008	5.5786	222.25	0.0017
30	5.5882	221.87	0.0026	5.5892	221.83	0.0006

Table S4a. TD-M062X/cc-pVDZ electron transitions of stable enol-keto forms (model **IIa** and **IIb**) of Cs symmetry. Excitation energies (E_{exc}) and corresponding wavelengths (λ) are in eV and nm, respectively. Oscillator strengths (*f*) are in atomic units.

	**IIa**			**IIb**		
**No.**	E_{exc}	λ	f	E_{exc}	λ	f
1	3.4748	356.81	1.8055	3.4587	358.47	1.8296
2	3.8497	322.07	0.0000	3.8483	322.18	0.0000
3	4.1591	298.10	0.0793	4.1184	301.05	0.0738
4	4.6104	268.92	0.0171	4.5929	269.95	0.0070
5	4.7916	258.75	0.0044	4.8002	258.29	0.0051
6	4.8666	254.77	0.0216	4.8317	256.61	0.0137
7	5.0135	247.30	0.0231	5.0015	247.89	0.0165
8	5.3497	231.76	0.0900	5.3718	230.80	0.0622
9	5.5719	222.52	0.1899	5.5277	224.30	0.3720
10	5.6098	221.01	0.0593	5.5572	223.10	0.1553
11	6.0137	206.17	0.0012	5.9882	207.05	0.0277
12	6.0801	203.92	0.1531	6.0573	204.68	0.0319
13	6.1353	202.08	0.0000	6.1469	201.7	0.0000
14	6.2088	199.69	0.0675	6.2071	199.74	0.0547
15	6.3234	196.07	0.0225	6.3200	196.18	0.0128
16	6.5197	190.17	0.0580	6.5663	188.82	0.1836
17	6.5856	188.27	0.0188	6.6041	187.74	0.0558
18	6.6356	186.85	0.1268	6.6724	185.82	0.0383
19	6.7794	182.88	0.1010	6.7260	184.34	0.2303
20	6.8286	181.57	0.0000	6.7949	182.47	0.0709
21	6.8569	180.82	0.2005	6.7962	182.43	0.0000
22	6.8738	180.37	0.0000	6.8000	182.33	0.0000
23	6.8836	180.12	0.0000	6.8264	181.62	0.0000
24	6.9245	179.05	0.2188	6.9381	178.7	0.0001
25	6.9817	177.58	0.0000	6.9722	177.83	0.0748
26	7.0341	176.26	0.0001	7.0263	176.46	0.1108
27	7.0846	175.01	0.0002	7.0431	176.04	0.0001
28	7.0979	174.68	0.0391	7.1747	172.81	0.0006
29	7.1940	172.34	0.0004	7.3142	169.51	0.0047
30	7.2539	170.92	0.0410	7.3779	168.05	0.1018

Table S4b. TD-B3LYP(SMD=water)/aug-cc-pVDZ//M062X/cc-pVDZ electron transitions of stable enol-keto forms (model **IIa** and **IIb**) of C_s symmetry. Excitation energies (E_{exc}) and wavelengths (λ) are in eV and nm, respectively. Oscillator strengths (*f*) are in atomic units.

	**IIa**			**IIb**		
**No.**	E_{exc}	λ	f	E_{exc}	λ	f
1	2.7314	453.93	1.6966	2.6990	459.37	1.6146
2	3.1498	393.63	0.0357	3.0832	402.13	0.0450
3	3.6410	340.52	0.0942	3.6318	341.39	0.2576
4	3.7230	333.02	0.0001	3.7182	333.45	0.0001
5	3.7431	331.23	0.0179	3.7864	327.44	0.0117
6	4.0306	307.61	0.0984	4.0429	306.67	0.0649
7	4.0776	304.06	0.0527	4.0791	303.95	0.0333
8	4.2868	289.23	0.1836	4.2620	290.91	0.1218
9	4.6683	265.59	0.0319	4.6170	268.54	0.0064
10	4.6947	264.09	0.0139	4.6566	266.26	0.0487
11	4.8975	253.16	0.0007	4.8858	253.77	0.0005
12	4.9254	251.72	0.0304	4.9138	252.32	0.0466
13	4.9553	250.21	0.0306	4.9462	250.67	0.0662
14	4.9805	248.94	0.0001	4.9524	250.35	0.0001
15	5.0398	246.01	0.0297	5.0599	245.03	0.1204
16	5.0848	243.83	0.0332	5.0832	243.91	0.0286
17	5.1173	242.28	0.0000	5.0850	243.82	0.0001
18	5.2015	238.36	0.0050	5.1639	240.10	0.0460
19	5.2658	235.45	0.0000	5.2117	237.90	0.0107
20	5.2788	234.87	0.0880	5.2739	235.09	0.0000
21	5.3175	233.16	0.0004	5.2828	234.70	0.2278
22	5.3197	233.07	0.0298	5.2877	234.48	0.0001
23	5.3358	232.36	0.0001	5.3079	233.58	0.0034
24	5.3678	230.98	0.0052	5.3709	230.85	0.0000
25	5.4098	229.18	0.0005	5.3732	230.74	0.0559
26	5.4396	227.93	0.0001	5.4245	228.56	0.0005
27	5.5264	224.35	0.0008	5.4945	225.65	0.0006
28	5.5988	221.45	0.0004	5.4998	225.43	0.0128
29	5.6105	220.99	0.1156	5.5633	222.86	0.0015
30	5.6776	218.37	0.0007	5.6369	219.95	0.0000

Table S5a. TD-M062X/cc-pVDZ electron transitions of stable enol-keto forms (model **IIa** and **IIb**) of C_1 symmetry. Excitation energies (E_{exc}) and corresponding wavelengths (λ) are in eV and nm, respectively. Oscillator strengths (*f*) are in atomic units.

	**IIa**			**IIb**		
**No.**	E_{exc}	λ	f	E_{exc}	λ	f
1	3.4457	359.82	1.4016	3.4238	362.12	1.4750
2	3.8839	319.23	0.0010	3.8916	318.60	0.0001
3	4.1773	296.81	0.1874	4.1488	298.84	0.1690
4	4.5828	270.54	0.0458	4.5733	271.10	0.0482
5	4.7972	258.45	0.0043	4.8097	257.78	0.0023
6	4.8375	256.30	0.0097	4.8431	256.00	0.0302
7	4.9122	252.40	0.0364	4.8970	253.19	0.0410
8	5.3070	233.62	0.0988	5.2805	234.80	0.0883
9	5.5886	221.85	0.0661	5.5343	224.03	0.2766
10	5.5995	221.42	0.3352	5.5855	221.97	0.0753
11	5.8358	212.46	0.0081	5.8800	210.86	0.0197
12	5.9897	207.00	0.0005	5.9933	206.87	0.0001
13	6.0879	203.66	0.1145	6.0713	204.21	0.0273
14	6.2418	198.64	0.0026	6.2096	199.67	0.0800
15	6.3511	195.22	0.0344	6.4099	193.42	0.1638
16	6.4488	192.26	0.0874	6.4782	191.39	0.1774
17	6.5464	189.39	0.0571	6.5463	189.39	0.0045
18	6.6402	186.72	0.0669	6.6152	187.42	0.0322
19	6.7240	184.39	0.2092	6.7443	183.84	0.0945
20	6.7499	183.68	0.0037	6.7669	183.22	0.1049
21	6.7886	182.64	0.0035	6.7750	183.00	0.0017
22	6.8044	182.21	0.0330	6.8202	181.79	0.0000
23	6.863	180.66	0.0005	6.8860	180.05	0.0319
24	6.9006	179.67	0.1838	6.8935	179.86	0.1186
25	6.9537	178.30	0.0037	6.8981	179.74	0.0043
26	7.0249	176.49	0.0074	7.0258	176.47	0.1173
27	7.0346	176.25	0.0431	7.0416	176.07	0.0047
28	7.1899	172.44	0.0025	7.1391	173.67	0.0602
29	7.2137	171.87	0.0648	7.2533	170.93	0.0001
30	7.2408	171.23	0.0030	7.3168	169.45	0.0296

Table S5b. TD-B3LYP(SMD=water)/aug-cc-pVDZ//M062X/cc-pVDZ electron transitions of stable enol-keto forms (model **IIa** and **IIb**) of C_1 symmetry. Excitation energies (E_{exc}) and wavelengths (λ) are in eV and nm, respectively. Oscillator strengths (*f*) are in atomic units.

	IIa			IIb		
**No.**	E_{exc}	λ	f	E_{exc}	λ	f
1	2.7385	452.74	1.3795	2.7308	454.02	1.4201
2	3.1719	390.88	0.0825	3.1561	392.85	0.1135
3	3.6641	338.38	0.0928	3.6527	339.43	0.1091
4	3.7808	327.93	0.0008	3.7784	328.14	0.0003
5	3.7914	327.01	0.0089	3.7996	326.31	0.0200
6	3.9312	315.38	0.0989	3.9442	314.35	0.0849
7	4.0750	304.25	0.1204	4.0485	306.25	0.0938
8	4.2006	295.16	0.1443	4.2015	295.10	0.1231
9	4.5845	270.44	0.0279	4.5944	269.86	0.0021
10	4.6767	265.11	0.0117	4.6589	266.13	0.0158
11	4.8042	258.08	0.1038	4.7989	258.36	0.0865
12	4.8927	253.40	0.0007	4.9003	253.01	0.0006
13	4.9635	249.79	0.0030	4.9479	250.58	0.0549
14	4.9647	249.73	0.0391	4.9562	250.16	0.0000
15	4.9985	248.04	0.0119	5.0415	245.93	0.1285
16	5.1050	242.87	0.0064	5.0991	243.15	0.0069
17	5.1141	242.44	0.0531	5.1162	242.34	0.0001
18	5.1667	239.97	0.0011	5.1577	240.39	0.0003
19	5.1808	239.31	0.0033	5.1623	240.17	0.0820
20	5.2093	238.01	0.0924	5.2386	236.67	0.0701
21	5.2645	235.51	0.0049	5.2773	234.94	0.0055
22	5.2865	234.53	0.0001	5.2943	234.18	0.0835
23	5.3182	233.13	0.1361	5.3014	233.87	0.0258
24	5.3257	232.80	0.0043	5.3164	233.21	0.0001
25	5.3879	230.12	0.0007	5.3761	230.62	0.0002
26	5.4375	228.02	0.0003	5.4542	227.32	0.0001
27	5.5110	224.98	0.0118	5.4898	225.85	0.0011
28	5.5267	224.34	0.0713	5.5347	224.01	0.0357
29	5.5718	222.52	0.0013	5.5727	222.48	0.0018
30	5.6452	219.63	0.0076	5.6564	219.19	0.0001

Table S6a. TD-M062X/cc-pVDZ electron transitions of stable anionic enolate forms (model **IIIa** and **IIIb**) of C_{2V} symmetry. Excitation energies (E_{exc}) and corresponding wavelengths (λ) are in eV and nm, respectively. Oscillator strengths (*f*) are in atomic units.

	**IIIa**			**IIIb**		
**No.**	E_{exc}	λ	f	E_{exc}	λ	f
1	2.9519	420.01	0.0000	2.9554	419.52	0.0000
2	3.0447	407.21	0.7231	3.0563	405.66	0.7882
3	3.6121	343.25	0.0000	3.6210	342.40	0.0000
4	3.6370	340.90	0.0082	3.6801	336.9	0.0038
5	4.4036	281.55	0.0007	4.3734	283.49	0.0165
6	4.4280	280.00	0.0493	4.3741	283.45	0.0067
7	4.5908	270.07	0.8164	4.6403	267.19	0.0000
8	4.6055	269.21	0.0000	4.6542	266.39	1.0123
9	4.7010	263.74	0.1511	4.7618	260.37	0.1714
10	5.0580	245.13	0.2510	4.9337	251.30	0.0527
11	5.0776	244.18	0.0019	4.9372	251.12	0.0050
12	5.2519	236.08	0.0000	5.3355	232.38	0.0001
13	5.4229	228.63	0.0841	5.4060	229.35	0.0000
14	5.4476	227.59	0.0000	5.4103	229.16	0.0000
15	5.4678	226.75	0.0000	5.4295	228.35	0.0978
16	5.5277	224.30	0.0011	5.7375	216.09	0.0170
17	5.7242	216.60	0.0000	5.7574	215.35	0.0103
18	5.7252	216.56	0.0210	5.7796	214.52	0.0000
19	5.7508	215.60	0.0020	5.8834	210.73	0.0027
20	5.8062	213.54	0.0000	6.0077	206.38	0.0000
21	6.0518	204.87	0.0003	6.0110	206.26	0.0000
22	6.0674	204.35	0.0000	6.0625	204.51	0.0000
23	6.0740	204.12	0.1188	6.0646	204.44	0.0000
24	6.0844	203.77	0.0995	6.1155	202.74	0.5108
25	6.1925	200.22	0.0014	6.1203	202.58	0.0901
26	6.2999	196.08	0.0031	6.3555	195.08	0.0362
27	6.3204	196.17	0.0000	6.4058	193.55	0.0673
28	6.4414	192.48	0.3802	6.4197	193.13	0.0183
29	6.4684	191.68	0.0166	6.4514	192.18	0.0000
30	6.5115	190.41	0.0000	6.4519	192.17	0.0000

Table S6b. TD-B3LYP(SMD=water)/aug-cc-pVDZ//M062X/cc-pVDZ electron transitions of stable anionic enolate forms (model **IIIa** and **IIIb**) of C_{2V} symmetry. Excitation energies (E_{exc}) and wavelengths are in eV and nm, respectively. Oscillator strengths (*f*) are in atomic units.

	IIIa			IIIb		
**No.**	E_{exc}	λ	f	E_{exc}	λ	f
1	3.0182	410.79	1.4935	3.0112	411.74	1.5070
2	3.2159	385.54	0.0000	3.2134	385.84	0.0000
3	3.5425	349.99	0.0729	3.4982	354.42	0.0827
4	3.7798	328.01	0.0120	3.7421	331.32	0.0002
5	3.9625	312.89	0.0700	4.0092	309.25	0.0789
6	4.0855	303.47	0.0000	4.0863	303.42	0.0000
7	4.2002	295.19	0.0021	4.2413	292.32	0.0247
8	4.2402	292.40	0.0536	4.2439	292.15	0.0111
9	4.3944	282.14	0.2954	4.4005	281.75	0.2445
10	4.4745	277.09	0.0000	4.4940	275.89	0.0000
11	4.5483	272.59	0.0597	4.5783	270.81	0.0378
12	4.6245	268.10	0.0964	4.5968	269.72	0.1945
13	4.6344	267.53	0.0003	4.6102	268.93	0.0186
14	4.7334	261.93	0.0040	4.6539	266.41	0.0004
15	4.7906	258.81	0.0000	4.7785	259.46	0.0000
16	4.8882	253.64	0.0014	4.8667	254.76	0.0017
17	5.0062	247.66	0.0038	4.9472	250.62	0.0503
18	5.0283	246.57	0.0053	4.9527	250.34	0.0007
19	5.0623	244.92	0.0001	5.0770	244.21	0.0041
20	5.1511	240.69	0.0000	5.1042	242.91	0.0000
21	5.1740	239.63	0.0000	5.1629	240.15	0.0000
22	5.1990	238.48	0.0010	5.1721	239.72	0.0546
23	5.2388	236.67	0.0622	5.2294	237.09	0.0028
24	5.2496	236.18	0.0023	5.2420	236.52	0.0000
25	5.2524	236.05	0.0000	5.2778	234.92	0.0048
26	5.2835	234.66	0.0089	5.2854	234.58	0.2609
27	5.3050	233.71	0.0001	5.3050	233.71	0.0034
28	5.3261	232.79	0.0103	5.3302	232.61	0.0095
29	5.3329	232.49	0.0025	5.3314	232.56	0.0170
30	5.3635	231.16	0.1855	5.3724	230.78	0.0000

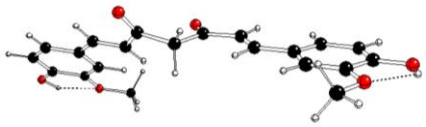

model **Ia**, C_2 group

(a)

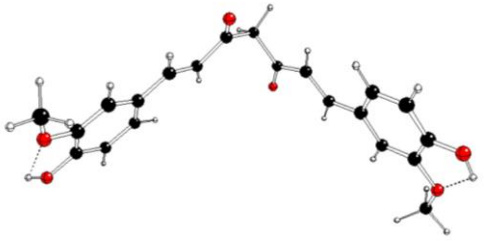

model **Ib**, C_2 group

(b)

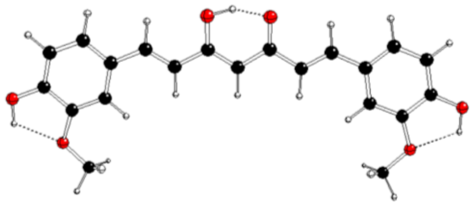

model **IIa**, C_s group

(c)

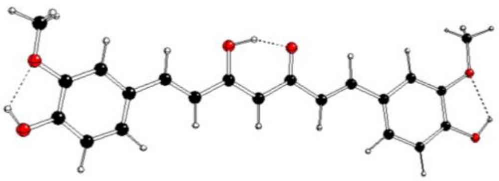

model **IIb**, C_s group

(d)

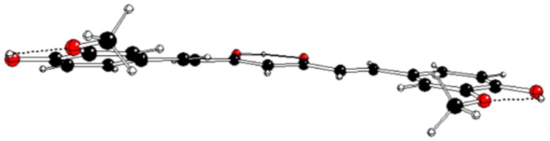

model **IIa**, C_1 group

(e)

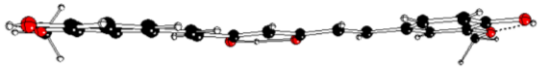

model **IIb**, C_1 group

(f)

Figure S1. M062X/cc-pVDZ optimized curcumin structures of C_1, C_2 and C_s groups of the neutral diketo forms (a, b), and neutral enol forms (c, d, e, f) (C – black, O – red, H – white).

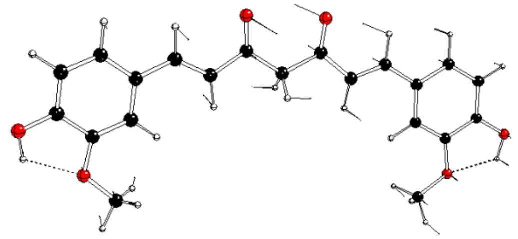

model **Ia**, C_{2v} group

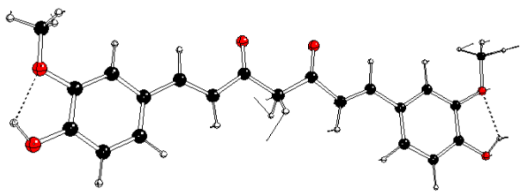

model **Ib**, C_{2v} group

Figure S2. Pseudo-JT active vibration of a_2 symmetry in curcumin keto-forms **Ia** (left) and **Ib** (right) of C_{2v} group (see Fig. S1 for atom notation).

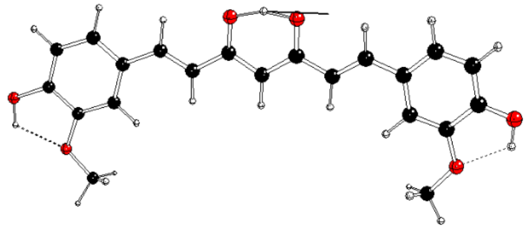

model **IIa**, C_{2v} group

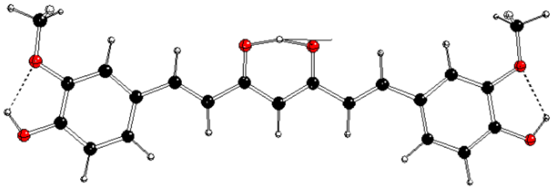

model **IIb**, C_{2v} group

Figure S3. Pseudo-JT active vibration of b_1 symmetry in curcumine enol-forms **IIa** (left) and **IIb** (right) of C_{2v} group (see Fig. S1 for atom notation).

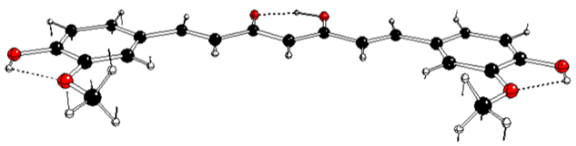

model **IIa**, C_s group

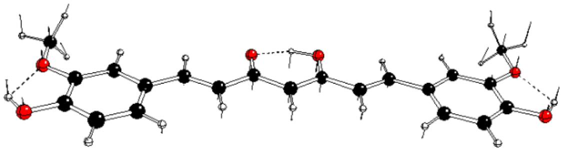

model **IIb**, C_s group

Figure S4. Pseudo-JT active vibration of a” symmetry in curcumine enol-forms **IIa** (left) and **IIb** (right) of C_s group (see Fig. S1 for atom notation).

REFERENCE:

[S1] Ceulemans, A.; Vanquickenborne, L. G. The Epikernel Principle. *Struct. Bonding*, 1989, **71**, 125. DOI: 10.1007/3-540-50775-2_4
